# Application of U/Th and ^40^Ar/^39^Ar Dating to Orgnac 3, a Late Acheulean and Early Middle Palaeolithic Site in Ardèche, France

**DOI:** 10.1371/journal.pone.0082394

**Published:** 2013-12-05

**Authors:** Véronique Michel, Guanjun Shen, Chuan-Chou Shen, Chung-Che Wu, Chrystèle Vérati, Sylvain Gallet, Marie-Hélène Moncel, Jean Combier, Samir Khatib, Michel Manetti

**Affiliations:** 1 Université Nice Sophia Antipolis, Campus Saint-Jean-d'Angély, SJA3 - CEPAM- UMR 7264 CNRS, Nice Cedex 4, France; 2 Géoazur, UMR7329, UNS-CNRS-IRD-OCA, Valbonne, France; 3 College of Geographical Sciences, Nanjing Normal University, Nanjing, China; 4 High-precision Mass Spectrometry and Environment Change Laboratory (HISPEC), Department of Geoscience, National Taiwan University N°1, Taipei, Taiwan ROC; 5 Département de Préhistoire, UMR 7194, MNHN, IPH, Paris, France; 6 CNRS, Macon, France; 7 Laboratoire Départemental de Préhistoire du Lazaret, Nice, France; 8 Université Nice Sophia Antipolis, Département Terre-Environnement-Espace, Parc Valrose, Nice Cedex 2, France; University of Oxford, United Kingdom

## Abstract

Refined radio-isotopic dating techniques have been applied to Orgnac 3, a Late Acheulean and Early Middle Palaeolithic site in France. Evidence of Levallois core technology appeared in level 4b in the middle of the sequence, became predominant in the upper horizons, and was best represented in uppermost level 1, making the site one of the oldest examples of Levallois technology. In our dating study, fourteen speleothem samples from levels 7, 6 and 5b, were U/Th-dated. Four pure calcite samples from the speleothem PL1 (levels 5b, 6) yield ages between 265 ± 4 (PL1-3) and 312 ± 15 (PL1-6) thousand years ago (ka). Three samples from the top of a second stalagmite, PL2, yield dates ranging from 288 ± 10 ka (PL2-1) to 298 ± 17 ka (PL2-3). Three samples from the base of PL2 (level 7) yield much younger U/Th dates between 267 and 283 ka. These dates show that the speleothems PL1 and PL2 are contemporaneous and formed during marine isotope stage (MIS) 9 and MIS 8. Volcanic minerals in level 2, the upper sequence, were dated by the ^40^Ar/^39^Ar method, giving a weighted mean of 302.9 ± 2.5 ka (2σ) and an inverse isochron age of 302.9 ± 5.9 ka (2σ). Both ^40^Ar/^39^Ar dating of volcanic sanidines and U/Th dating of relatively pure and dense cave calcites are known to be well established. The first parallel application of the two geochronometers to Orgnac 3 yields generally consistent results, which point to the reliability of the two methods. The difference between their age results is discussed.

## Introduction

**Figure 1:**
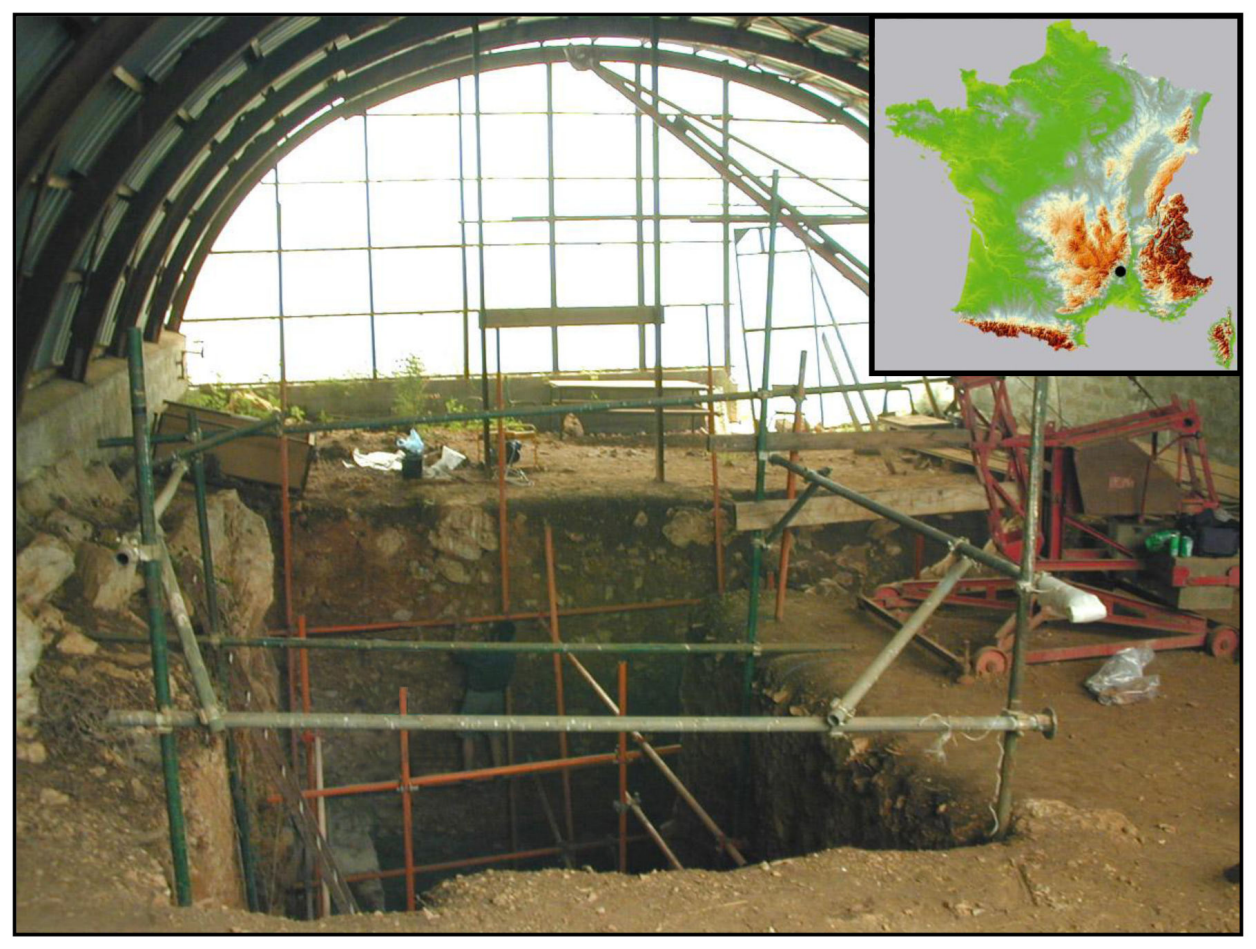
Localization of Orgnac 3 in France and an overview of the site.

The Orgnac 3 site is located at a place called Mattecarlinque, at an altitude of 320 m, on the southwest fringe of an Urgonian karstic plateau (lower Cretaceous), in southern Ardèche, central France [1-4] (Figure **1**). The site was initially a cave with human settlement, later changed into a rock shelter, and finally became an open-air site [5] (Figure **1**). The depositional sequence is 11m thick. The lower archaeological levels (8 to 4a) were deposited in a cave context while the upper levels 2-1 were accumulated in an open-air environment. Seven hominin teeth, in levels 6, 5b and 5a, assigned to *Homo heidelbergensis* [6], about 50,000 stone artefacts and abundant mammal fossils have been discovered [1]. Bone assemblages indicate the predominance of carnivores in lower levels (8 and 7), cervids in levels 6-5a, bovids in levels 4b-3 and equids in upper levels 2 and 1. According to biostratigraphical correlation, the lower levels (8 to 3) are attributed to the Middle Pleistocene (MIS 9) and the upper levels 2 and 1 to the late Middle Pleistocene (MIS 8). Levallois debitage, marking the beginning of the Middle Palaeolithic, appears in the middle strata and becomes predominant at the top of the sequence, producing changes in tool kits, raw material procurement and subsistence strategies [1,5]. A reliable chronology for this site is thus particularly important for understanding human cultural evolution and the onset of Neandertal culture. The aim of this study is to refine the age intervals using high-precision U/Th dating on intercalated speleothems and the ^40^Ar/^39^Ar method on well-preserved volcanic minerals in the upper strata. Note that both of these methods are considered as reliable for establishing a temporal frame for human evolution. 

### Stratigraphy, biostratigraphy and lithic industry

The depositional sequence can be divided into four major stratigraphic units (I, II, III, IV) and 12 sedimentary levels (Ia to Ie, IIa and IIb, IIIa to IIIc, IVa and IVb) [7], with 10 archaeological (1, 2, 3, 4a, 4b, 5a, 5b, 6, 7 and 8) and 3 hominin fossil-bearing (6, 5b, 5a) levels [2,5,8] (see table in [9]). 

**Figure 2:**
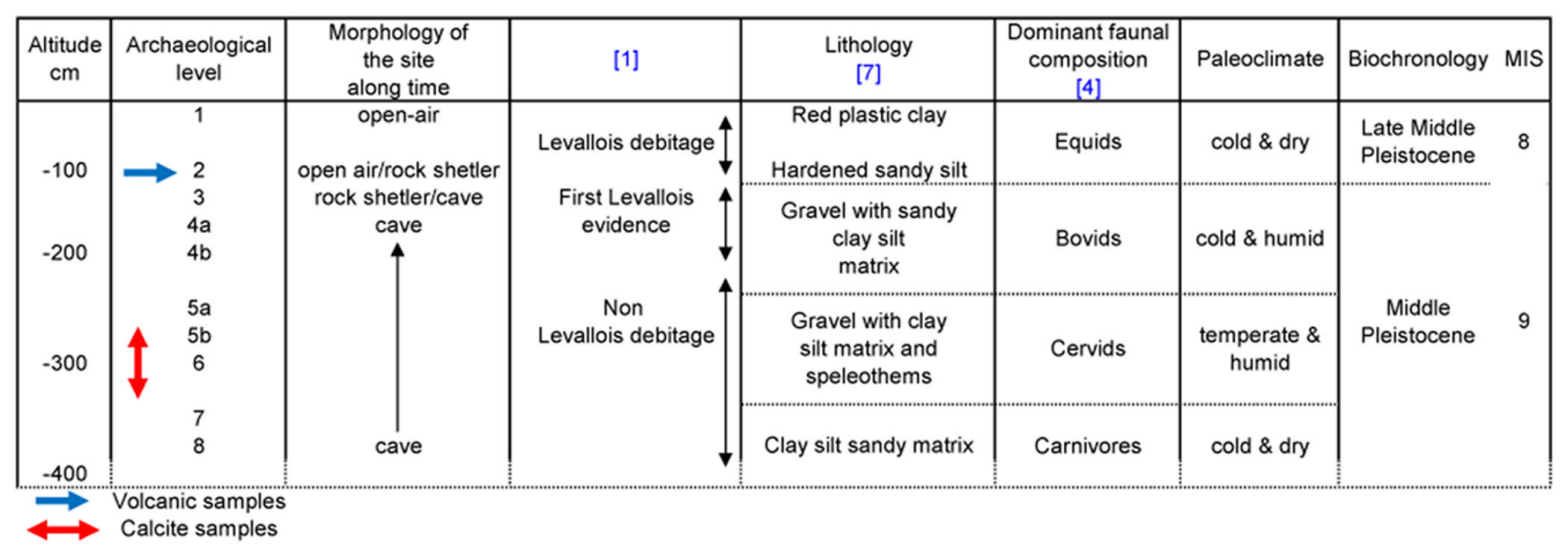
Archaeological levels, stratigraphic levels of Orgnac 3, dominant faunal composition, paleoclimate and biostratigraphy.

The lowermost unit I includes five levels (Ia - Ie) composed of bedded-sandy-clay with angular gravels [7,9]. This unit, containing mainly carnivore and reindeer remains (archaeological levels 8, 7), including small sized *Canis lupus*, *Crocuta crocuta spelaea*, *Ursus thibetanus*, *Vulpes vulpes*, *Panthera* (*Leo*) *spelaea*, *Ursus deningeri*, *Ursus arctos*, appears to have been deposited under a generally cold climate [1,4] (Figure **2**). Unit II, divided into three archaeological levels (6, 5b and 5a) with a preponderance of *Cervus elaphus, Dama clactoniana, Capreolus sussenbornensis* and *Sus scrofa* fossils, is composed of silty deposits with eroded gravels, large fallen blocks and speleothem formations [7], corresponding to a humid and temperate climate (MIS9, [1]) (Figure **2**). Further up, unit III is composed of three sedimentary levels (IIIa to IIIc) of clayey sand with angular gravels and blocks, with abundant *Bovidae* fossils, corresponding to a cool and humid climate. Three archaeological horizons (4b, 4a, 3) can be identified (Figure **2**). The uppermost unit IV, including two archaeological levels (1 and 2), is composed of clayey deposits with some gravels [7]. This unit marks the last human occupation of the site, and contains predominantly *Equus steinheimensis* remains, corresponding to a cooler climate and an open landscape [1] (Figure **2**).

Evidence of the emergence of Middle Palaeolithic-type behavior can be observed throughout the depositional sequence with the development of long and complex flaking reduction sequences. In the lower levels (7 to 5a), debitage is mainly represented by centripetal cores. In the middle levels (4b and 4a), the first evidence of Levallois cores can be observed. In the top levels (3 to 1), Levallois cores on flakes are dominant. Two groups of levels may be distinguished by observing the flake-tool kit; levels 8-3 (with a broader diversity of flake-tools) and levels 2-1 (containing a majority of scrapers with thinner retouch). The shaping reduction sequences are limited throughout the whole sequence (bifaces and pebble tools). In levels 2 and 1, the frequency of bifaces is very low (less than 1%), and these are mainly bifacial tools with few removals. Various criteria related to technical behavior and subsistence strategy patterns indicate gradual changes over time towards Middle Palaeolithic-type behavior from the bottom to the top of the sequence.

### Previous chronological studies

**Figure 3:**
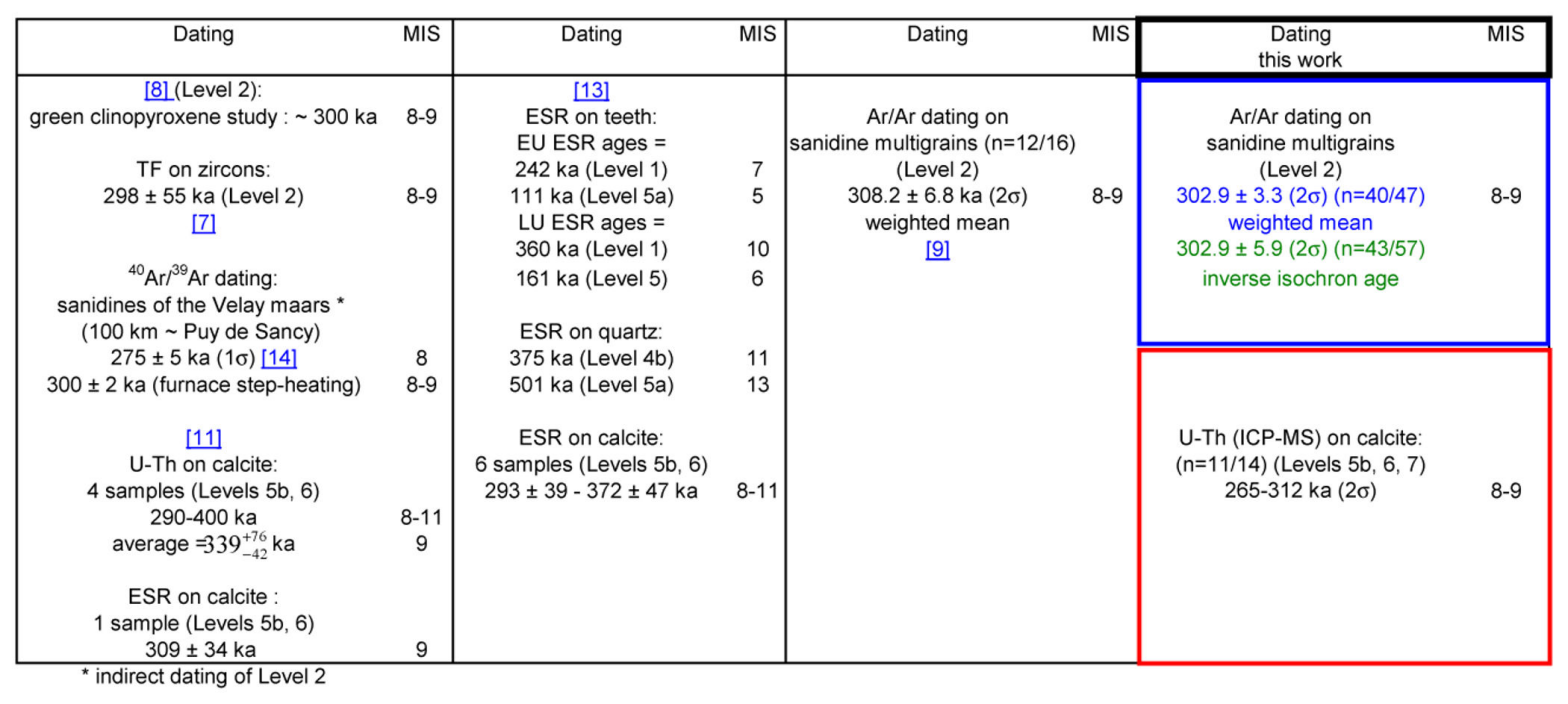
Summary of previously obtained ages and ages obtained in this work.

**Figure 4:**
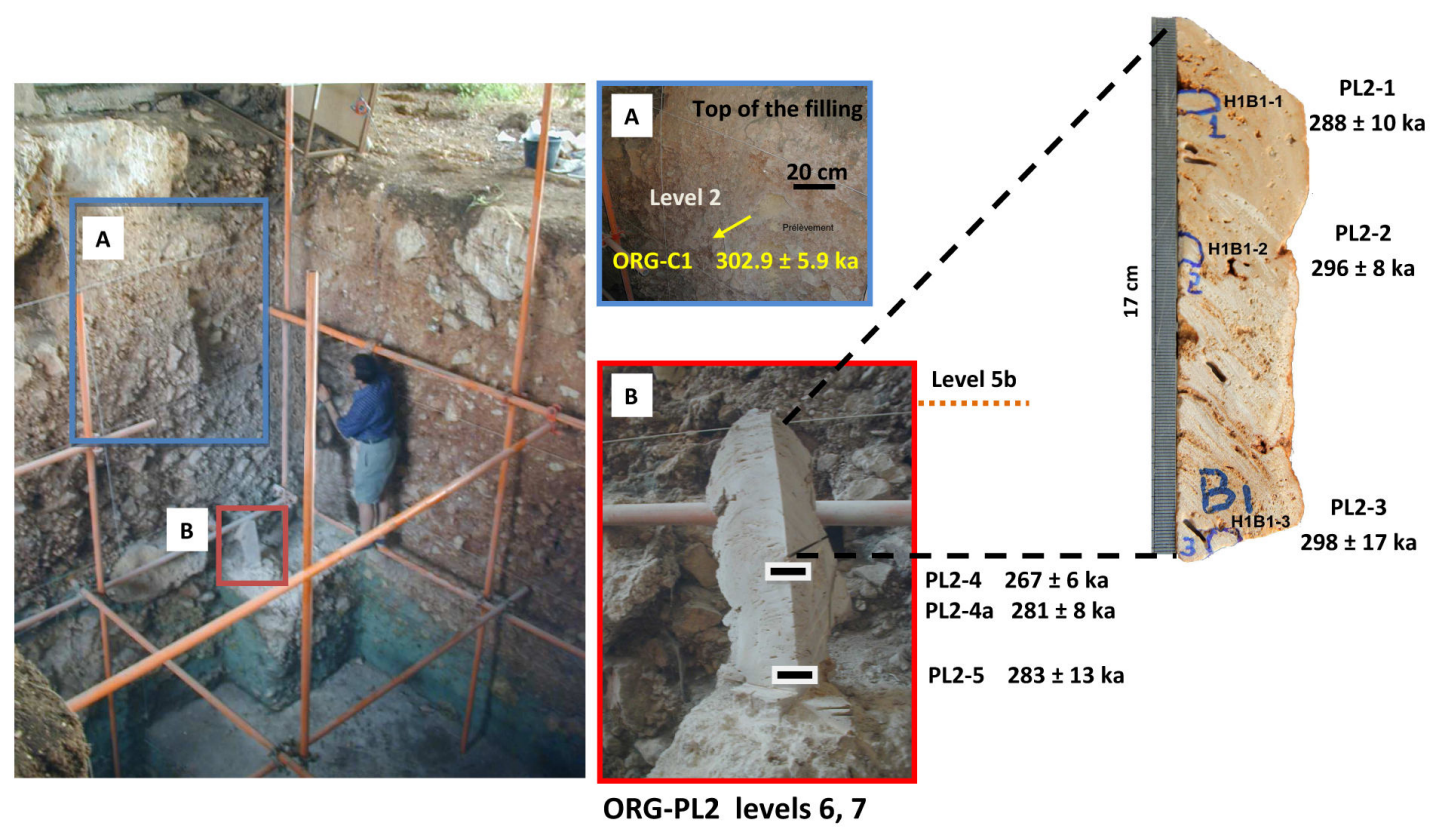
Position of level 2 and of levels 7 and 6 at Orgnac 3. (A) of volcanic inclusion ORG-C1 in the upper unit level 2 and (B) of calcite samples number PL2-1 to PL2-5 (levels 7 and 6).

The first dating of Orgnac 3 was carried out in 1985 [10]. Four speleothem samples from archaeological levels 7 and 6, and between levels 6 and 5b were dated with the alpha spectrometric U/Th method (Figure **3**). Based on the results obtained, the author proposed that the mean age of four age results $339_{-42}^{+76}$ ka, should be taken as the best age estimate for the speleothem formations. One of the four calcite samples was also analyzed by the electron spin resonance (ESR) method, yielding an age of 309 ± 34 ka [11]. At about the same time, Debard and Pastre [8] described and analyzed fallout volcanic ashes in the upper archaeological level 2, which is composed of lightly brown silty sand [7]. The volcanic ashes there are yellowish inclusions several tens of centimeters in diameter (an example of such an inclusion is given in Figure **4A**, marked as ORG-C1). The authors [8] extracted well- preserved angular green pyroxenes, which are characteristic of one of the last eruptions of the Puy de Sancy volcano (Mont-Dore, Massif Central, France) [12]. They proposed an age of about 300 ka for the upper level 2. With the fission track (FT) technique, Khatib [7] analyzed 22 zircons from volcanic ashes from the same level, obtaining an age of 298 ± 55 ka (Figure **3**). Masaoudi [13] presented the results of U/Th and ESR dating of bones and teeth and ESR dating of calcite and quartz samples from different levels (Figure **3**). The measured dates are overdispersed, and do not conform well to the stratigraphy. Recently ^40^,Ar/^39^Ar dating was carried out on 16 sets of sanidine grains [9]. Four of them yield ages too old to be acceptable because of contamination by inherited K feldspar grains, while the remaining 12 ages are between 276 and 326 ka with a weighted mean of 308.2 ± 6.8 ka. Roger et al. [14] considered that the tephra layers at the Praclaux and du Bouchet maars (French Massif Central) and at Orgnac 3 all came from the eruption of the Sancy volcano centre. Based on the weighted mean of ^40^Ar/^39^Ar dates on a series of sanidines from the Praclaux and Bouchet lakes, they assigned an age of 275 ± 5 ka to the Sancy eruption. Recently, Nomade et al. [12] recalculated the age according to ACR-2 at 1.193 Ma and obtained an age of 279 ± 5 ka. Roger et al. [14] also determined a step-heating plateau age of 300 ± 2 ka from sanidine grain populations (Figure **3**). For their experiments, the neutron fluence (J) was monitored with a biotite Bern B4B with an age of 17.25 Ma and a sanidine Draz with an age of 24.99 Ma [14], recently recalibrated to 25.42 Ma [15]. 

## Results

### U/Th dating

**Figure 5:**
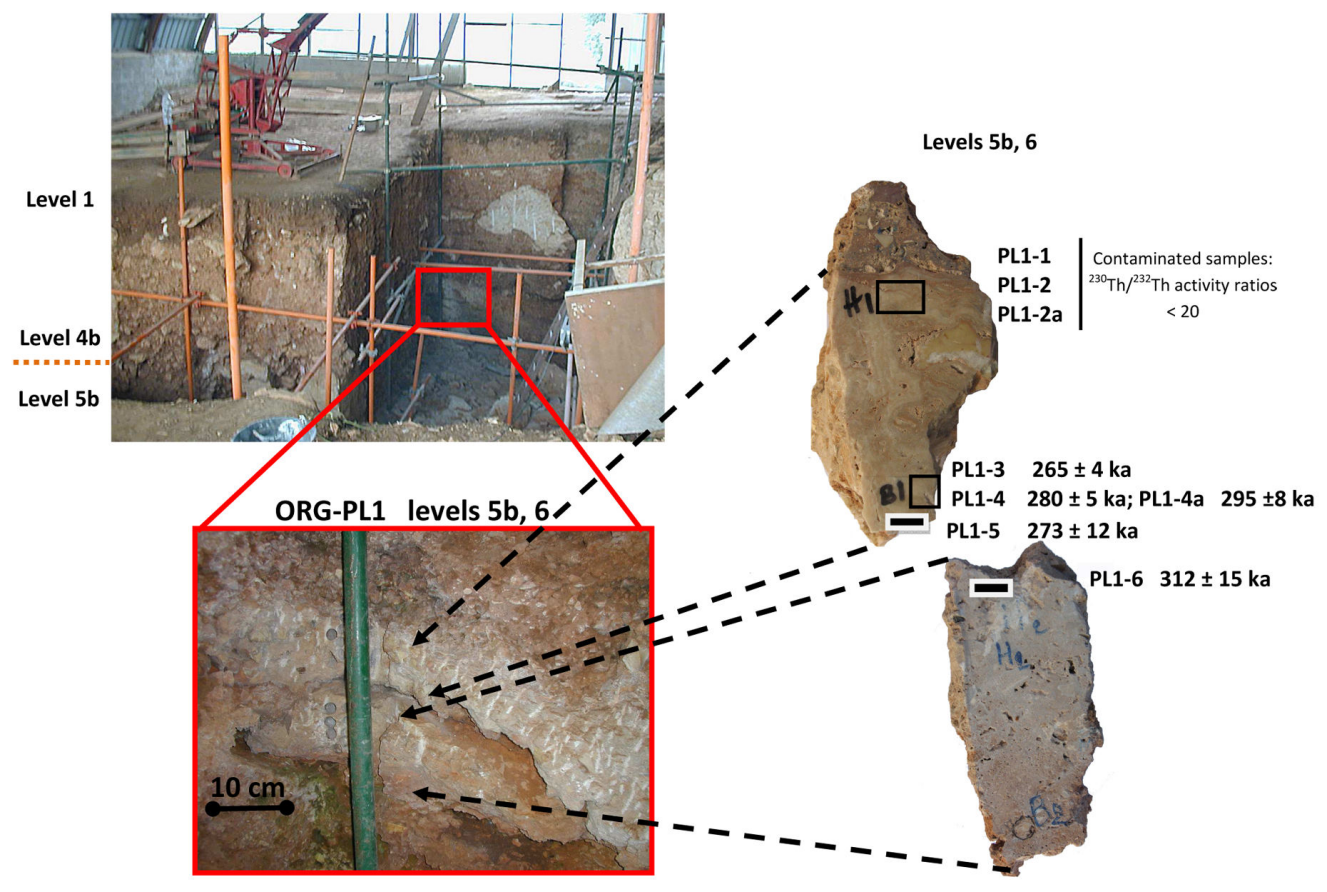
Position of speleothem samples number PL1-1 to PL1-6 (levels 6 and 5b).

**Figure 6:**
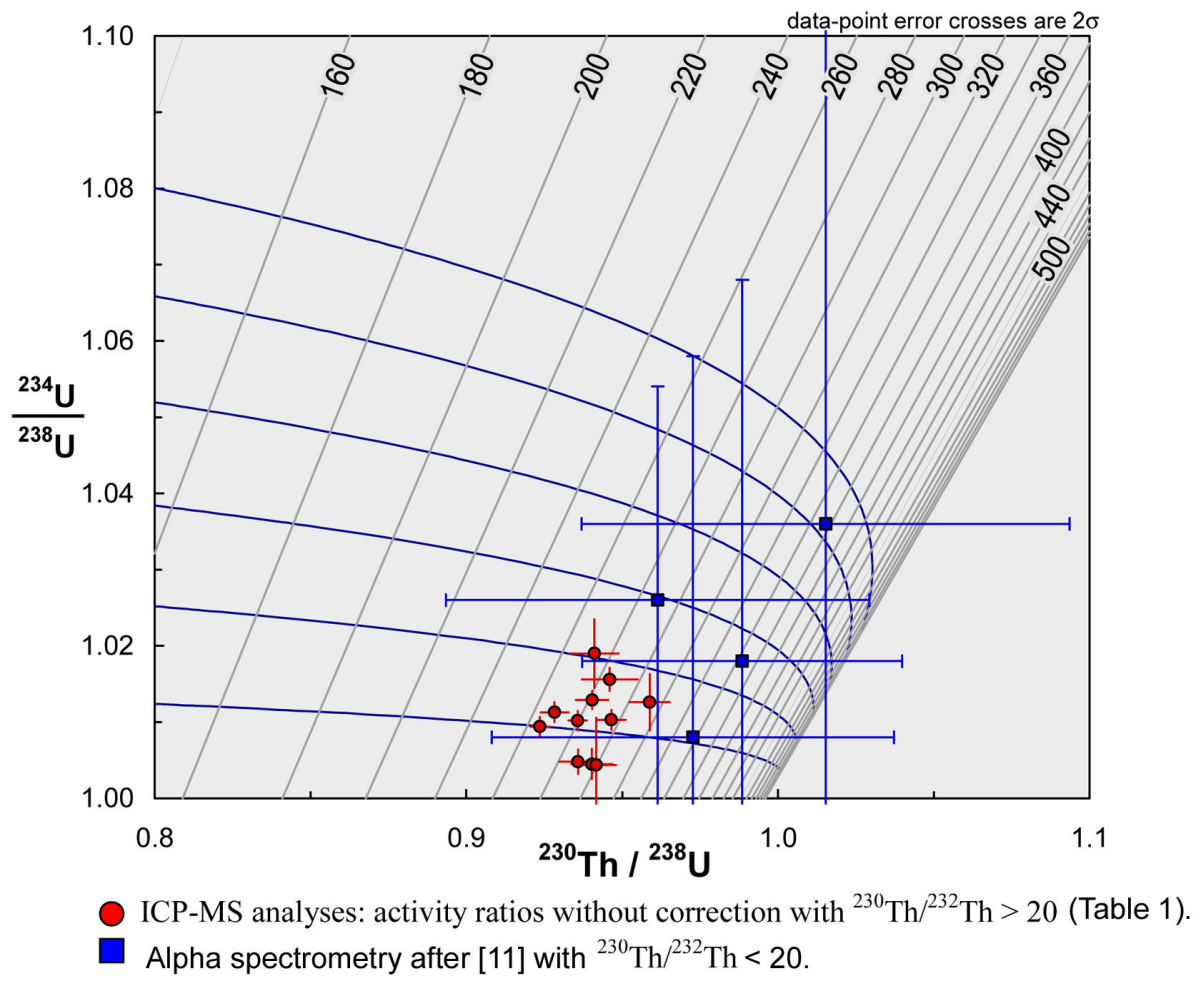
U/Th diagram obtained from Isoplot program [21] with quotation of previous alpha spectrometer data at 2σ level.

Isotopic measurements of the fourteen speleothem samples are presented in Table **1** and in Figures **4**, **5**. The uranium content ranges from 66 ppb to 148 ppb, and thorium from 250 to 32,495 ppt. Samples are mostly free from detrital contamination as indicated by ^230^Th/^232^Th activity ratios higher than 20. Only three samples PL1-1, PL1-2 and PL1-2a have low [^230^Th/^232^Th] activity ratios (10.0, 10.9 and 17.6 respectively) indicating contamination by detrital materials (Table **1**, Figure **5**). Note that PL1-2 and PL1-2a are taken from exactly the same position. But compared with PL1-2a (with a [^230^Th/^232^Th] activity ratio of 17.6), the more contaminated PL1-2 (10.9) gives a significantly younger age result (218 ka << 275 ka, Table **1**). For this, we tend to consider that the samples with a low [^230^Th/^232^Th] activity ratio may have undergone metamorphism leading to underestimated age results. Therefore these three (ICP-MS) U/Th ages were excluded (Table **1**, Figure **5**). The precision of ICP-MS isotopic ratio measurements is much better in comparison with the previous alpha measurements, as shown in Figure **6**. For the base of the upper part of the first speleothem PL1, U/Th dates range from 265 ± 4 ka (PL1-3) and 295 ± 8 ka (PL1-4a) (levels 5b-6) (Figure **5**. The top of the lower part of this speleothem yielded a date of 312 ± 15 ka (PL1-6) (Figure **5**). For the second stalagmite PL2 (Figure **4**), U/Th dates range from 281 ± 8 ka (PL2-4a) to 298 ± 17 ka (PL2-3), except for the date of 267 ± 6 ka for sample PL2-4. This youngest date is significantly different from the date, 281 ± 8 ka, of the coeval sample PL2-4a and the mean date of 289 ± 16 ka for the other 5 dates of this speleothem. This abnormally young date is probably biased by post-depositional diagenesis and is thus excluded from this study. U/Th ages indicate that both speleothems from levels 7-6-5b range from 265 ka (marine isotope stage 8, MIS 8) to 312 ka (MIS 9). 

**Table 1:** Uranium and thorium isotopic compositions and U/Th ages of Orgnac 3 samples by ICP-MS, Thermo Electron Neptune and Element II at NTU.

**Sample**	**Levels**	**^238^U**		**^232^Th**		**δ^234^U**		**[^234^U/^238^U]**		**[^230^Th/^238^U]**		**[^230^Th/^232^Th]**		**Age (ka)**		**Age (ka)**		**δ ^234^U_initial_**	
**N°**		**(ppb) ^*a*^**		**(ppt)**		**measured ^*a*^**		**Activity**		**activity ^*c*^**		**activity ^*d*^**		**uncorrected**		**corrected ^*c*,*e*^**		**corrected^*b*^**	
PL1-1 *	5b, 6	111.80	± 0.12	31,003	± 193	13.9	± 1.4	1.0139	± 0.0014	0.906	± 0.010	9.99	± 0.13	241.030	± 10.194	n.d.			
PL1-2 *	5b, 6	131.86	± 0.17	32,495	± 186	15.0	± 1.7	1.0150	± 0.0017	0.881	± 0.010	10.93	± 0.14	218.171	± 8.051	n.d.			
PL1-2a *	5b, 6	118.19	± 0.11	19,260	± 80	12.9	± 1.2	1.0129	± 0.0012	0.9356	± 0.0073	17.56	± 0.15	275.609	± 9.928	n.d.			
PL1-3 *	5b, 6	65.967	± 0.066	866.3	± 3.6	9.4	± 1.3	1.0094	± 0.0013	0.9236	± 0.0031	214.9	± 1.1	265.701	± 4.224	265.360	± 4.225	19.8	± 2.7
PL1-4 *	5b, 6	68.555	± 0.073	761.9	± 3.3	10.2	± 1.3	1.0102	± 0.0013	0.9357	± 0.0030	257.3	± 1.3	280.218	± 4.657	279.930	± 4.653	22.4	± 2.9
PL1-4a *	5b, 6	77.193	± 0.078	3448	± 8.2	10.3	± 1.3	1.0103	± 0.0013	0.9465	± 0.0047	64.76	± 0.35	296.260	± 8.054	295.099	± 8.053	23.7	± 3.0
PL1-5 **	5b, 6	67.12	± 0.15	249.6	± 2.3	19.0	± 4.5	1.0190	± 0.0045	0.9411	± 0.0078	773.7	± 9.4	273.354	± 12.165	273.260	± 12.154	41.2	± 9.9
PL1-6 **	5b, 6	81.14	± 0.19	3038.8	± 9.4	12.6	± 3.7	1.0126	± 0.0037	0.9588	± 0.0065	78.24	± 0.55	312.521	± 14.863	311.556	± 14.734	30.4	± 9.1
PL2-1 *	6, 7	104.94	± 0.12	7239	± 26	4.8	± 1.6	1.0048	± 0.0016	0.9358	± 0.0060	41.64	± 0.30	290.039	± 9.786	288.218	± 9.795	10.7	± 3.6
PL2-2 *	6, 7	109.88	± 0.18	4985	± 21	4.5	± 2.0	1.0045	± 0.0020	0.9403	± 0.0066	63.36	± 0.51	297.817	± 11.744	296.623	± 11.676	10.3	± 4.6
PL2-3 *	6, 7	123.98	± 0.31	8365	± 31	4.4	± 6.2	1.0044	± 0.0062	0.9417	± 0.0064	42.65	± 0.31	300.149	± 16.959	298.368	± 16.759	10	± 14
PL2-4 *	6, 7	125.70	± 0.16	5360	± 14	11.3	± 1.3	1.0113	± 0.0013	0.9283	± 0.0044	66.54	± 0.35	268.755	± 5.839	267.648	± 5.885	24.0	± 2.9
PL2-4a *	6, 7	147.96	± 0.16	7667	± 24	12.9	± 1.2	1.0129	± 0.0012	0.9404	± 0.0052	55.46	± 0.34	282.307	± 7.564	280.965	± 7.592	28.4	± 2.7
PL2-5 *	6, 7	137.96	± 0.18	15,698	± 87	15.6	± 1.5	1.1056	± 0.0015	0.9460	± 0.0090	25.41	± 0.28	285.678	± 13.303	282.729	± 13.289	34.5	± 3.5

ICP-MS model:* MC-ICP-MS [16]; ** SF-ICP-MS [17]. Analytical errors are 2σ of the mean. **^*a*^**[^238^U] = [^235^U] x 137.818 (±0.65‰) [18]; δ^234^U = ([^234^U/^238^U]_activity_ - 1) x 1000. **^*b*^**δ ^234^U_initial_ corrected was calculated based on ^230^Th age (T), i.e., δ ^234^U_initial_ = δ^234^U_measured_ *X* e^λ234*T^, and T is corrected age. **^*c*^**[^230^Th/^238^U]_activity_ = 1 - e^-λ230T^ + (δ^234^U_measured_/1000)[λ_230_/(λ_230_ - λ_234_)](1 - e^-(λ230 - λ234) T^), where *T* is the age. Decay constants are 9.1705 x 10^-6^ yr **^*-*^** ^1^ for ^230^Th, 2.8221 x 10^-6^ yr **^*-*^** ^1^ for ^234^U [19], and 1.55125 x 10^-10^ yr **^*-*^** ^1^ for ^238^U [20]. **^*d*^**The degree of detrital ^230^Th contamination is indicated by the [^230^Th/^232^Th] activity ratio. **^*e*^**Age corrections for samples were calculated using an estimated activity ^230^Th/^232^Th ratio of 0.74 (± 100%). Those are the values for a material at secular equilibrium, with the crustal ^232^Th/^238^U value of 3.8 with 100% uncertainty. n.d. = not determined: these samples are excluded because they have [^230^Th/^232^Th] activity ratio < 20.

### ^40^Ar/^39^Ar dating

**Figure 7:**
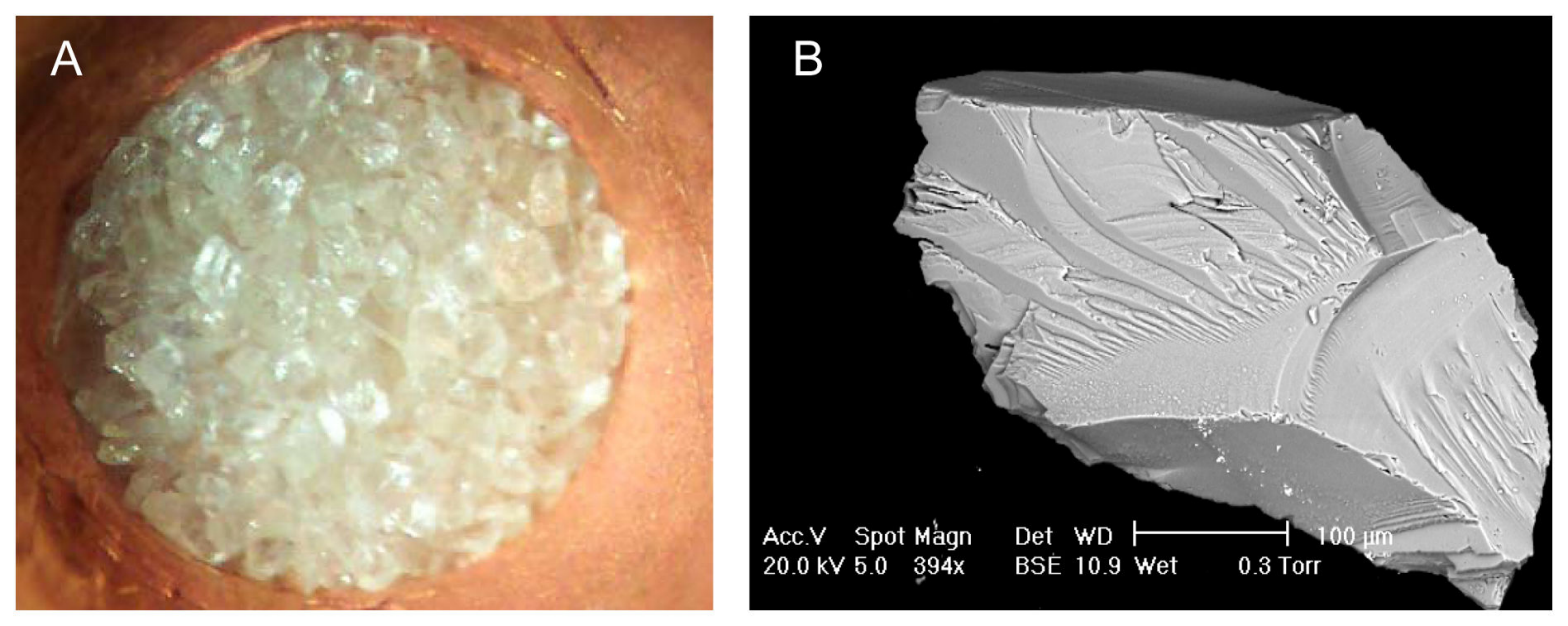
Sanidine minerals at Orgnac 3. (A) About 150 grains (200-300 μm) for a total fusion analysis. (B) Orgnac sanidine (SEM). The minerals are sharp-edged and unweathered.

Fifty-seven sanidine grain populations were analyzed with the total-fusion method (Figure **7**). In order to obtain precise data, 50-150 sanidine grains were analyzed for each measurement ^40^Ar/^39^Ar ages with counting errors at 2σ are shown in Tables **2**, **3**, **4**. Ten samples with determined ages older than 550 ka are most likely contaminated by inherited K feldspar grains. These samples, representing 17.5% of the results, were not taken into account (Tables **2**, **3**, **4**). The abnormally old age results may be explained by the presence of old minerals, such as plagioclases or sanidines, extracted from the base rock during the Sancy volcano eruption.

**Table 2:** Analytical ^40^Ar/^39^Ar data summary of samples from Orgnac 3 (level 2) (Lab. # K346-2 to K346-25) (see footnotes in Table 4).

**Sample**	**Lab N°**	**^40^Ar (moles)**	**^40^Ar (V)**	**± 1σ**	**^39^Ar (V)**	**± 1σ**	**^38^Ar (V)**	**± 1σ**	**^37^Ar (V)**	**± 1σ**	**^36^Ar (V)**	**± 1σ**	^40^Ar*/ ^*39*^Ark	**± 1σ**	%^40^Ar*	**Age (ka)**	**± 2σ**
Blank	B346-1		0.002313	0.000035	0.000015	0.000007	0.000005	0.000004	0.000027	0.000010	0.000020	0.000008					
Sanidine	K346-2	2.115E-15	0.108072	0.000216	0.141057	0.000136	0.001791	0.000006	0.000588	0.000008	0.000071	0.000004	0.632142	0.02091	84.5	284.5	± 18.8
Sanidine	K346-3	2.173E-15	0.110942	0.000229	0.140594	0.000286	0.001775	0.000014	0.000544	0.000013	0.000075	0.000003	0.647114	0.02040	83.9	291.3	± 18.4
Blank	B346-2		0.002252	0.000018	0.000087	0.000011	0.000024	0.000004	0.000100	0.000008	0.000020	0.000002					
Sanidine	K346-4	2.945E-15	0.149503	0.000302	0.194349	0.000535	0.002434	0.000018	0.000841	0.000008	0.000075	0.000005	0.662450	0.01189	87.6	298.2	± 10.7
Sanidine	K346-5	1.494E-15	0.076928	0.000127	0.096335	0.000231	0.001209	0.000003	0.000396	0.000010	0.000057	0.000004	0.650727	0.01581	84.0	292.9	± 14.2
Blank	B346-3		0.002355	0.000032	0.000025	0.000006	0.000015	0.000003	0.000080	0.000006	0.000024	0.000005					
Sanidine	K346-6	2.292E-15	0.116975	0.000269	0.138666	0.000386	0.001734	0.000017	0.000610	0.000011	0.000093	0.000006	0.675911	0.01892	81.5	304.2	± 17.0
Sanidine	K346-7	2.895E-15	0.147108	0.000353	0.187460	0.000480	0.002333	0.000018	0.000801	0.000014	0.000088	0.000006	0.665610	0.01500	85.9	299.6	± 13.5
Sanidine	K346-8	2.747E-15	0.139715	0.000270	0.173531	0.000453	0.002168	0.000020	0.000715	0.000014	0.000078	0.000011	0.695159	0.02180	87.5	312.9	± 19.6
Blank	B346-4		0.002738	0.000036	0.000118	0.000012	0.000015	0.000005	0.000083	0.000006	0.000032	0.000006					
Sanidine	K346-9	1.877E-15	0.096595	0.000231	0.122468	0.000704	0.001550	0.000012	0.000532	0.000011	0.000055	0.000004	0.705738	0.01904	91.7	317.6	± 17.1
Sanidine	K346-10	7.795E-15	0.392509	0.000757	0.146539	0.000310	0.001824	0.000014	0.000561	0.000016	0.000067	0.000007	2.592475	0.03262	97.0	* 1166.5	± 29.3
Sanidine	K346-11	1.779E-15	0.091699	0.000254	0.113530	0.000501	0.001420	0.000011	0.000498	0.000009	0.000042	0.000011	0.752419	0.03250	95.6	338.6	± 29.3
Blank	B346-5		0.002653	0.000042	0.000066	0.000009	0.000034	0.000006	0.000073	0.000007	0.000023	0.000005					
Sanidine	K346-13	5.045E-15	0.254926	0.000566	0.122390	0.000286	0.001519	0.000019	0.000513	0.000013	0.000038	0.000004	2.025957	0.02596	97.9	* 911.7	± 23.4
Sanidine	K346-14	2.521E-15	0.128700	0.000210	0.167022	0.000624	0.002135	0.000023	0.000689	0.000009	0.000076	0.000005	0.656814	0.01464	86.7	295.6	± 13.2
Blank	B346-6		0.002735	0.000057	0.000121	0.000015	0.000004	0.000004	0.000083	0.000007	0.000023	0.000006					
Sanidine	K346-15	1.808E-15	0.093128	0.000139	0.117389	0.000291	0.001483	0.000011	0.000506	0.000009	0.000060	0.000005	0.672288	0.02119	86.9	302.6	± 19.1
Sanidine	K346-16	4.945E-15	0.249972	0.000430	0.150912	0.000306	0.001929	0.000023	0.000669	0.000010	0.000083	0.000005	1.520405	0.02179	92.4	* 684.2	± 19.6
Blank	B346-7		0.002167	0.000037	0.000007	0.000005	0.000006	0.000007	0.000002	0.000013	0.000008	0.000007					
Sanidine	K346-17	2.823E-15	0.143316	0.000319	0.188726	0.001019	0.002437	0.000030	0.000718	0.000020	0.000054	0.000006	0.675740	0.01605	89.6	304.1	± 14.4
Sanidine	K346-18	2.977E-15	0.150993	0.000352	0.185636	0.000486	0.002305	0.000023	0.000689	0.000006	0.000125	0.000007	0.617218	0.01747	76.3	277.8	± 15.7
Sanidine	K346-19	2.081E-15	0.106218	0.000369	0.137491	0.000368	0.001733	0.000016	0.000489	0.000010	0.000055	0.000005	0.656114	0.01964	85.9	295.3	± 17.7
Blank	B348-8		0.002223	0.000070	0.000085	0.000006	0.000006	0.000002	0.000019	0.000010	0.000028	0.000004					
Sanidine	K346-20	9.422E-16	0.049331	0.000268	0.059375	0.000406	0.000737	0.000015	0.000219	0.000016	0.000033	0.000006	0.764799	0.03956	95.4	344.2	± 35.6
Sanidine	K346-21	1.448E-15	0.074611	0.000405	0.098834	0.000584	0.001271	0.000019	0.000381	0.000014	0.000019	0.000006	0.756301	0.02333	100.0	340.4	± 21.0
Sanidine	K346-22	1.307E-15	0.067582	0.000219	0.081598	0.000374	0.001018	0.000022	0.000365	0.000027	0.000049	0.000008	0.723949	0.03254	89.5	325.8	± 29.3
Blank	B348-9		0.002367	0.000045	0.000103	0.000014	0.000001	0.000005	0.000008	0.000008	0.000022	0.000005					
Sanidine	K346-23	1.748E-15	0.089756	0.000521	0.121182	0.000521	0.001544	0.000012	0.000503	0.000026	0.000044	0.000004	0.666709	0.01799	91.6	300.1	± 16.2
Sanidine	K346-24	3.376E-15	0.171187	0.000866	0.222005	0.001938	0.002736	0.000041	0.000851	0.000034	0.000064	0.000014	0.704840	0.02199	91.8	317.2	± 19.8
Sanidine	K346-25	2.165E-15	0.110596	0.000539	0.147713	0.001002	0.001817	0.000018	0.000551	0.000019	0.000057	0.000008	0.662120	0.02052	89.5	298.0	± 18.5

**Table 3:** Analytical ^40^Ar/^39^Ar data summary of samples from Orgnac 3 (level 2) (Lab. # K346-26 to K346-46) (see footnotes in Table 4).

**Sample**	**Lab N°**	**^40^Ar (moles)**	**^40^Ar (V)**	**± 1σ**	**^39^Ar (V)**	**± 1σ**	**^38^Ar (V)**	**± 1σ**	**^37^Ar (V)**	**± 1σ**	**^36^Ar (V)**	**± 1σ**	^40^Ar*/ ^*39*^Ark	**± 1σ**	%^40^Ar*	**Age (ka)**	**± 2σ**
Blank	B346-10		0.002361	0.000047	0.000028	0.000029	0.000011	0.000009	0.000067	0.000009	0.000024	0.000003					
Sanidine	K346-26	3.198E-15	0.162277	0.000387	0.214743	0.000511	0.002706	0.000024	0.000806	0.000026	0.000068	0.000005	0.680414	0.01141	90.9	306.2	± 10.3
Sanidine	K346-27	2.179E-15	0.111330	0.000756	0.137578	0.001254	0.001714	0.000014	0.000539	0.000025	0.000066	0.000003	0.698425	0.01474	87.7	314.3	± 13.3
Sanidine	K346-28	2.470E-15	0.125845	0.000414	0.161947	0.000626	0.002039	0.000017	0.000579	0.000003	0.000065	0.000006	0.684112	0.01430	89.3	307.9	± 12.9
Blank	B346-11		0.002483	0.000049	0.000113	0.000018	0.000016	0.000001	0.000050	0.000005	0.000033	0.000004					
Sanidine	K346-29	2.707E-15	0.137846	0.000338	0.178340	0.000334	0.002251	0.000010	0.000671	0.000011	0.000073	0.000005	0.688158	0.01327	90.2	309.7	± 11.9
Sanidine	K346-30	3.475E-15	0.176218	0.000635	0.113913	0.000442	0.001428	0.000017	0.000434	0.000012	0.000062	0.000007	1.448867	0.02793	94.5	* 652.0	± 25.1
Sanidine	K346-31	1.663E-15	0.085623	0.000132	0.113371	0.000335	0.001434	0.000012	0.000417	0.000009	0.000046	0.000003	0.694006	0.01579	94.1	312.4	± 14.2
Blank	B346-12		0.002367	0.000028	0.000074	0.000009	0.000006	0.000006	0.000099	0.000007	0.000026	0.000004					
Sanidine	K346-32	1.387E-15	0.071727	0.000131	0.090758	0.000218	0.001152	0.000012	0.000376	0.000006	0.000059	0.000007	0.653553	0.02616	85.0	294.2	± 23.5
Sanidine	K346-33	2.194E-15	0.112063	0.000576	0.149094	0.000488	0.001838	0.000012	0.000526	0.000021	0.000050	0.000003	0.684570	0.01209	92.6	308.1	± 10.9
Sanidine	K346-34	9.348E-15	0.469763	0.000905	0.152557	0.000362	0.001854	0.000017	0.000527	0.000019	0.000016	0.000008	3.091944	0.03611	100.0	* 1391.2	± 32.5
Sanidine	K346-35	1.760E-15	0.090375	0.000178	0.112439	0.000315	0.001427	0.000011	0.000437	0.000011	0.000074	0.000008	0.653960	0.02400	83.1	294.3	± 21.6
Blank	B351-1		0.002154	0.000027	0.000041	0.000012	0.000016	0.000005	0.000093	0.000006	0.000034	0.000004					
Sanidine	K346-36	3.477E-15	0.175985	0.000298	0.200637	0.000339	0.002552	0.000016	0.000628	0.000010	0.000115	0.000004	0.741072	0.01250	85.3	333.5	± 11.3
Blank	B352-2		0.001857	0.000025	0.000017	0.000009	0.000006	0.000003	0.000042	0.000009	0.000017	0.000005					
Sanidine	K346-37	3.217E-14	1.610252	0.001652	0.255068	0.000265	0.003020	0.000028	0.000619	0.000019	0.000073	0.000011	6.429203	0.06657	100.0	* 2891.6	± 59.8
Blank	B352-4		0.002173	0.000034	0.000056	0.000009	0.000003	0.000001	0.000059	0.000016	0.000024	0.000007					
Sanidine	K346-38	3.771E-15	0.190742	0.000559	0.238406	0.000913	0.003049	0.000020	0.000840	0.000020	0.000138	0.000011	0.645660	0.01841	81.3	290.6	± 16.6
Sanidine	K346-39	2.099E-15	0.107113	0.000214	0.138810	0.000293	0.001706	0.000016	0.000526	0.000013	0.000060	0.000006	0.673945	0.02059	88.7	303.3	± 18.5
Sanidine	K346-40	4.508E-15	0.227598	0.000490	0.280507	0.000910	0.003477	0.000026	0.000927	0.000006	0.000136	0.000011	0.680678	0.01578	84.3	306.4	± 14.2
Blank	B352-5		0.002127	0.000044	0.000013	0.000007	0.000005	0.000003	0.000026	0.000010	0.000017	0.000004					
Sanidine	K346-41	6.205E-15	0.312391	0.000748	0.163983	0.000531	0.002034	0.000022	0.000587	0.000012	0.000043	0.000007	1.847349	0.02435	97.1	* 831.3	± 21.9
Sanidine	K346-42	3.830E-15	0.193622	0.000438	0.243991	0.000709	0.003072	0.000025	0.000766	0.000009	0.000143	0.000003	0.628780	0.01091	79.7	283.0	± 9.8
Sanidine	K346-43	6.402E-15	0.322223	0.000847	0.246832	0.000603	0.003049	0.000029	0.000808	0.000022	0.000074	0.000007	1.227794	0.01672	94.1	* 552.6	± 15.0
Sanidine	K346-44	9.969E-15	0.500560	0.001361	0.288374	0.000738	0.003560	0.000024	0.000855	0.000006	0.000047	0.000007	1.698798	0.01990	97.7	* 764.5	± 17.9
Blank	B352-6		0.002406	0.000082	0.000190	0.000013	0.000014	0.000003	0.000080	0.000007	0.000020	0.000003					
Sanidine	K346-45	2.750E-15	0.139888	0.000310	0.182741	0.000483	0.002297	0.000010	0.000599	0.000020	0.000065	0.000008	0.677190	0.01613	89.4	304.8	± 14.5
Sanidine	K346-46	4.457E-15	0.225240	0.000492	0.292985	0.000816	0.003653	0.000026	0.000967	0.000018	0.000110	0.000008	0.667034	0.01170	87.2	300.2	± 10.5

**Table 4:** Analytical ^40^Ar/^39^Ar data summary of samples from Orgnac 3 (level 2) (Lab. # K346-47 to K346-59).

**Sample**	**Lab N°**	**^40^Ar (moles)**	**^40^Ar (V)**	**± 1σ**	**^39^Ar (V)**	**± 1σ**	**^38^Ar (V)**	**± 1σ**	**^37^Ar (V)**	**± 1σ**	**^36^Ar (V)**	**± 1σ**	^40^Ar*/ ^*39*^Ark	**± 1σ**	%^40^Ar*	**Age (ka)**	**± 2σ**
Blank	B352-7		0.002193	0.000045	0.000109	0.000014	0.000008	0.000004	0.000047	0.000005	0.000018	0.000006					
Sanidine	K346-47	4.373E-15	0.220828	0.000595	0.290423	0.000865	0.003608	0.000028	0.000928	0.000005	0.000097	0.000006	0.669352	0.01130	88.4	301.3	± 10.2
Sanidine	K346-48	4.525E-15	0.228425	0.000507	0.289917	0.000966	0.003643	0.000019	0.000951	0.000014	0.000112	0.000007	0.682064	0.01231	86.9	307.0	± 11.1
Sanidine	K346-49	6.081E-15	0.306248	0.000477	0.392298	0.000710	0.004864	0.000028	0.001224	0.000015	0.000127	0.000013	0.690350	0.01339	88.5	310.7	± 12.1
Blank	B352-8		0.002708	0.000090	0.000321	0.000044	0.000009	0.000005	0.000048	0.000008	0.000030	0.000004					
Sanidine	K346-50	1.151E-14	0.578434	0.002794	0.234564	0.000506	0.002873	0.000043	0.000724	0.000012	0.000028	0.000012	2.465336	0.03239	99.7	* 1109.3	± 29.1
Sanidine	K346-51	7.706E-15	0.388023	0.000485	0.434919	0.000896	0.005444	0.000038	0.001339	0.000013	0.000226	0.000007	0.750915	0.01092	84.2	338.0	± 9.8
Blank	B352-9		0.001994	0.000024	0.000010	0.000002	0.000007	0.000002	0.000044	0.000010	0.000023	0.000003					
Sanidine	K346-52	3.339E-15	0.168946	0.000396	0.218876	0.000589	0.002741	0.000025	0.000659	0.000013	0.000065	0.000009	0.703869	0.01471	91.5	316.8	± 13.2
Sanidine	K346-53	5.034E-15	0.253687	0.000565	0.330173	0.000809	0.004142	0.000029	0.000999	0.000012	0.000122	0.000008	0.672855	0.01101	87.5	302.8	± 9.9
Sanidine	K346-54	4.783E-15	0.241125	0.000506	0.314938	0.000699	0.003949	0.000028	0.000874	0.000013	0.000122	0.000006	0.665573	0.01000	86.9	299.6	± 9.0
Blank	B346-10		0.002195	0.000032	0.000190	0.000025	0.000022	0.000004	0.000052	0.000009	0.000016	0.000006					
Sanidine	K346-55	2.157E-15	0.110050	0.000317	0.145868	0.000478	0.001833	0.000017	0.000465	0.000016	0.000053	0.000008	0.664665	0.02223	89.0	299.2	± 20.0
Sanidine	K346-56	3.659E-15	0.185140	0.000488	0.233405	0.000886	0.002934	0.000018	0.000694	0.000014	0.000135	0.000008	0.634325	0.01605	80.2	285.5	± 14.4
Sanidine	K346-57	3.618E-15	0.183086	0.000384	0.234072	0.000553	0.002915	0.000026	0.000678	0.000016	0.000113	0.000008	0.650638	0.01518	83.4	292.8	± 13.7
Blank	B352-11		0.002521	0.000022	0.000103	0.000012	0.000008	0.000004	0.000053	0.000004	0.000030	0.000002					
Sanidine	K346-58	2.756E-15	0.140314	0.000411	0.177705	0.000617	0.002234	0.000022	0.000543	0.000013	0.000098	0.000007	0.662433	0.01411	84.7	298.1	± 12.7
Sanidine	K346-59	2.124E-15	0.108721	0.000217	0.139853	0.000317	0.001745	0.000018	0.000438	0.000011	0.000064	0.000008	0.687444	0.01959	89.7	309.4	± 17.6

* 10 analyses are excluded because these samples are presumed to be contaminated by older feldspar. Each analysis is the total laser fusion of about 50-150 sanidine grains of 200-300 μmn (~ 1.3 mg). Samples were irradiated for 40 min, with Cd shielding in 5C position at McMaster Nuclear Reactor. Heating = 120s. J factor is 0.0002495 ± 0.0000012 for the Alder Creek sanidine (1.194 ± 0.004 Ma, [22]), used as flux monitor. The spectrometer sensitivity is average 2.0 E-14 moles/V. Data are presented following [23] recommendations and calculations are determined by using the ArArCALC-software [24]. Mass discrimination, monitored by analyses of air pipette volume was 0.9979 per aum for lab # k346-2 to k346-5, 1.0037 for lab # k346-6 to k346-16, 1.0088 for lab # k346-17 to k346-25, 1.0047 for lab # k346-26 to k346-35, 1.0026 for lab # k346-36, 1.0044 for lab # k346-37 to k346-40, 1.0056 for lab # k346-41 to k346-51, 1.0082 for lab # k346-52 to k346-59. The (**^*40*^**Ar/^36^Ar) atmospheric argon ratio used is 298.56 ± 0.31 [30]. Interfering isotope production ratios: (**^*40*^**Ar/^39^Ar)_k_=0.0085 ± 0.0002 ; (**^*38*^**Ar/^39^Ar)_k_=0.0120 ± 0.0002 ; (**^*39*^**Ar/^37^Ar)_Ca_ = 0.00073± 0.00003 ; (**^*38*^**Ar/^37^Ar)_Ca_=0.006 ± 0.005 ; (**^*36*^**Ar/^37^Ar)_Ca_=0.000282 ± 0.000003 ^36^;Cl/^38^Cl = 316 ± 3.

Note that if 10 out of 57 samples are heavily contaminated, unless only one “inherited K feldspar” grain is enough to cause an abnormally old result, we should consider the possibility that the rest may be more or less contaminated.

**Figure 8:**
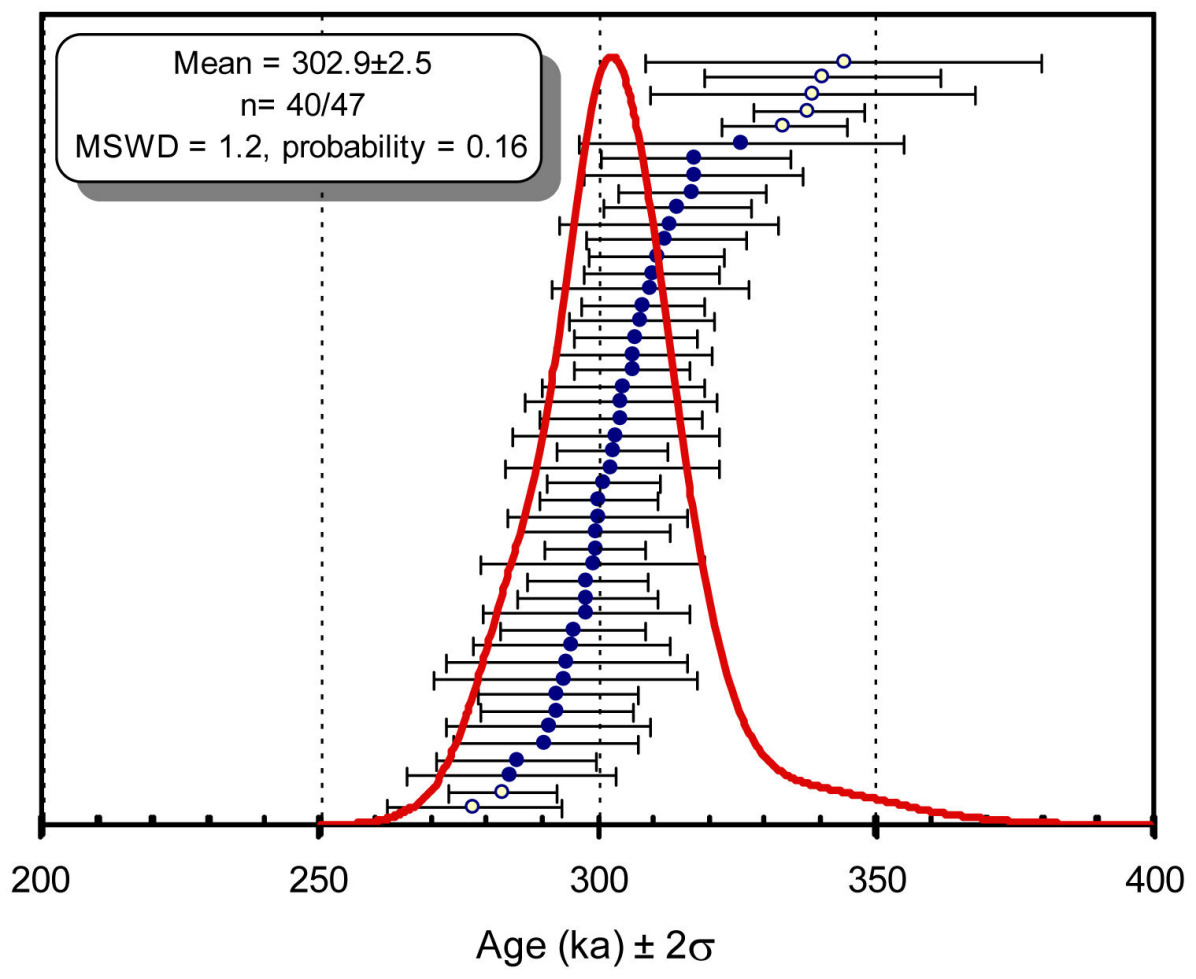
^40^Ar/^39^Ar ages distribution of Orgnac samples. The ^40^Ar/^39^Ar Orgnac ages are quoted at 2σ level with an age distribution (n=40/47) reported by a probability density distribution using Isoplot [21] (7 open circles are rejected). The weighted mean of 302.9 ± 2.5 ka (2σ, n=40/47, MSDW=1.2, P=0.16).

Forty-six dates, ranging from 283 to 344 ka, are associated with low atmospheric contamination (featuring over 79.7% of radiogenic argon, % ^40^Ar*). Another sample, K346-18 suffers from high atmospheric contamination (> 23.7% i.e. % ^40^Ar*<76.3, Tables **2**, **3**, **4**), yielding a young age (277.8 ka). Forty of the 47 dates yield an age distribution displayed by a probability density distribution (using Isoplot software, Figure **8**). The dominant mode of the distribution is centered at 302.9 ± 2.9 ka (2σ, n=40/47, MSDW=1.2, P=0.16) (Figure **8**). This age distribution is better than previous results [9]. In consequence, the weighted mean age of level 2 at Orgnac 3 is 302.9 ± 2.9 ka, corresponding to the transition from MIS 9 to MIS 8.

**Figure 9:**
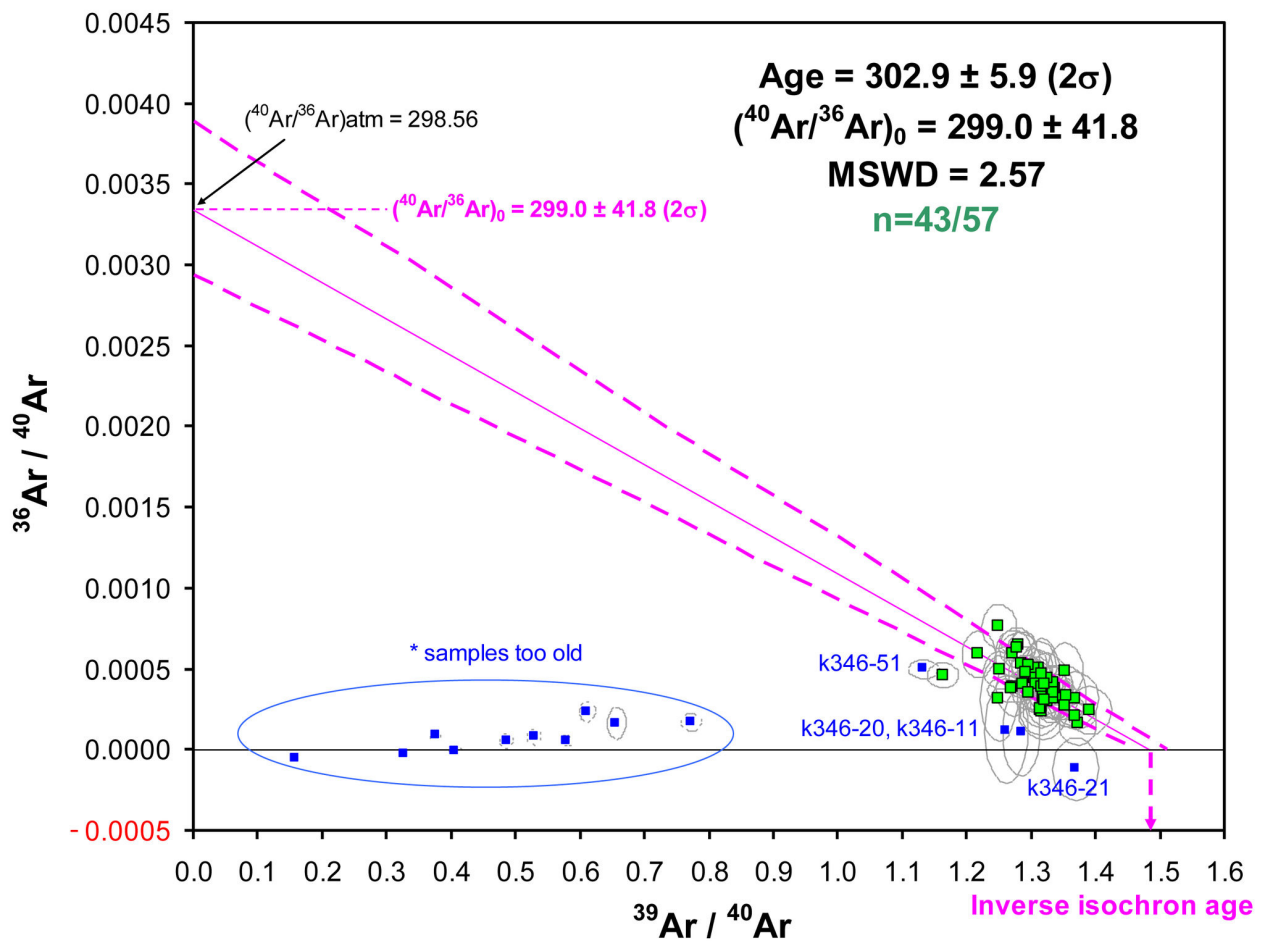
^36^Ar/^40^Ar vs ^39^Ar/^40^Ar inverse isochron diagram for all the data (n=57) with ellipse error at 2σ excluding 14 samples (blue squares): 1) *samples too old (quoted with * in Table 2) (n=10); 2) K346-20, K346-11 with a too high ^39^Ar/^36^Ar ratio and K346-21 with a negative ^36^Ar/^40^Ar ratio because ^36^Ar was underestimated; 3) K346-51 was eliminated otherwise the MSWD is 3.2 and the atmospheric ratio is not reached.

The measurement of ^36^Ar in ^40^Ar/^39^Ar dating allows for the plotting of the inverse isochron graph. The results are a series of data points ranging from pure atmospheric argon to pure radiogenic argon. A regression line through these data points forms an inverse isochron and the point at which the isochron intercepts with the x-axis yields the ^39^Ar/^40^Ar* of the samples and therefore the age. In Figure **9**, a regression line was plotted using ^36^Ar/^40^Ar and ^39^Ar/^40^Ar ratios (n=43/57), excluding 14 samples. Ten contaminated samples, K346-20 and K346-11 with high ^39^Ar/^36^Ar ratios, K346-21 with a negative ^36^Ar/^40^Ar ratio and K346-51 were eliminated in order to attain the atmospheric ratio (298.56; [25]) (Figure **9**). In these conditions, the atmospheric ratio obtained (^40^Ar/^36^Ar)_0_ is 299.0 ± 41.8 (2σ) and the intercept inverse isochron age is 302.9 ± 5.9 ka (2σ) (MSWD=2.57). This is in agreement with the weighted mean of 302.9 ± 2.9 ka (2σ) (Figure **8**). 

## Discussion

### U/Th and ^40^Ar/^39^Ar dating comparison

**Figure 10:**
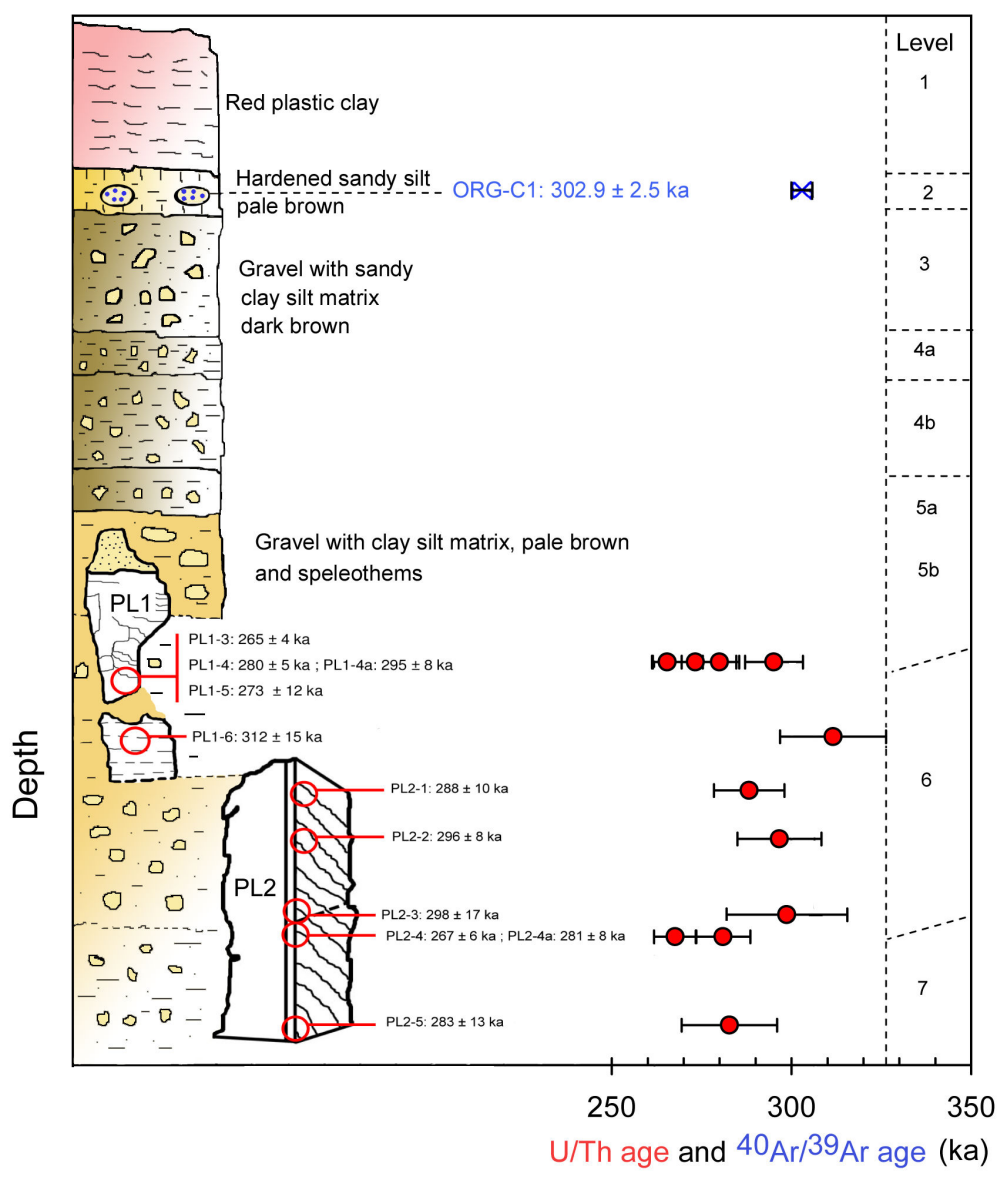
Stratigraphic positions of speleothem samples and volcanic minerals at Orgnac 3 with corresponding U/Th and ^40^Ar/^39^Ar dates.

The ^40^Ar/^39^Ar weighted mean age of 302.9 ± 2.9 ka and the inverse isochron age of 302.9 ± 5.9 ka are older than the upper limit of the measured U/Th age interval 265 to 312 ka (Figure **10**). The ^40^Ar/^39^Ar apparent ages determined in the higher level 2, should be younger than the U/Th apparent ages obtained in the median levels. This small difference in age (about 30 ka, which is not a major difference but is nonetheless statistically significant), could be explained by a slight contamination of sanidine grains or by a minor excess of ^40^Ar. Another hypothesis which may explain the age difference between the two methods may be that the volcanic minerals were transported to the site tens of thousand years after the Sancy eruption. In order to improve our ^40^Ar/^39^Ar dating results, we have tentatively used the step-heating method to highlight a possible ^40^Ar excess. In spite of the presence of excess ^40^Ar incorporated in minerals during crystallization [26], Renne et al. [27] have demonstrated in other studies with this method that a sanidine sample less than 2000 years old can be dated with 5% precision. However, for Orgnac 3, this step-heating method, using a VG 3600 mass spectrometer with a Daly detector, required a significant amount of material (up to 1,000 grains, 200 μm) which considerably increased the proportion of inherited K feldspar grains and thus increased the probability of an unreliable apparent age. 

**Figure 11:**
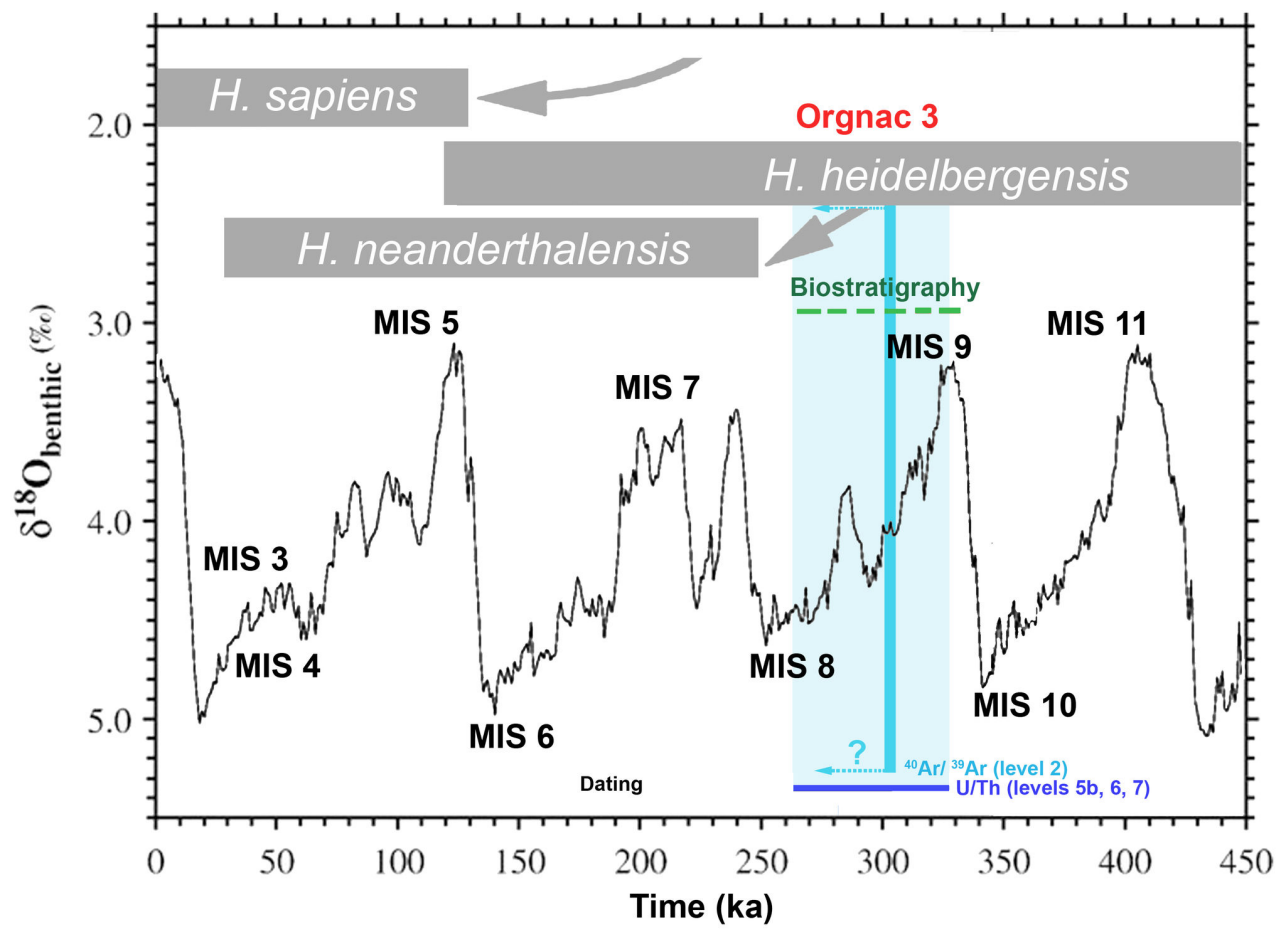
MIS and Orgnac infilling after U/Th and ^40^Ar/^39^Ar dating. Stacked δ^18^O record of benthic foraminifera from [28] after [29] with modifications. The shaded vertical envelope (± 2σ) shows the occurrence of the Orgnac infilling after U/Th and ^40^Ar/^39^Ar dating, close to the transition between MIS 8 and MIS 9.

In conclusion, taking into account our total fusion multigrain analyses, U/Th dates and errors, it seems reasonable to conclude that the Orgnac infilling is contemporaneous with MIS 9 and 8 (Figure **11**). The U-series date of 265 ka may mark a minimum age for the level 5b, when the Levallois flaking technique began to appear at the site (Figures **2**, **5** and **10**). This date is concordant with the biostratigraphical pattern which attributes levels 2 and 1 to MIS8 [1]. 

Thus, according to our U/Th ages, preliminary ^40^Ar/^39^Ar and the comparison between the two dating methods, Orgnac 3 is one of the oldest sites with the systematic use of Levallois knapping. As evidenced in [5,1], this temporal framework indicates the emergence of new technological behavior in southern France and Europe during MIS 8. Standardized core technology such as Levallois knapping can be observed in a few well-dated European sites close to the limit between MIS 9 and MIS 8, such as La Micoque (L2/3) (France), Gran Dolina (TD11/10), Bolomor (Spain) and la Baume Bonne (France) attributed to MIS 8 [1].

## Conclusion

For the first time, U/Th and ^40^Ar/^39^Ar dating methods have been applied together with greater precision than in previous studies for dating a Middle Pleistocene site. The ^40^Ar/^39^Ar dating gives a weighted mean age of 302.9 ± 2.9 ka (2σ) for upper level 2 of the Orgnac infilling while the U/Th method yields an age range of 265-312 ka for middle levels 7-6-5b. The age results from the two dating methods are generally consistent, which underlines their reliability. On the other hand, the difference between them is statistically significant taking into account the stratigraphical location of the samples. There are two possible explanations for an older ^40^Ar/^39^Ar age of 302.9 ± 2.9 ka (2σ). The first is that the volcanic minerals were transported to the site tens of thousand years after the Sancy eruption. The other possibility is that the analyzed sanidine grain populations have been systematically contaminated by inherited K feldspar grains. To check for the second hypothesis, it is necessary to carry out ^40^Ar/^39^Ar dating on single grains. We will soon carry out such research in order to add to the results reported in this paper. For this reason, here we only emphasize the U-series age of 265 ± 4 ka for level 5b, which attributes a minimal timeframe to the appearance of Levallois flaking at the site. Moreover, all our new U/Th ages suggest that the Orgnac 3 site lies within the 320-260 ka time range for the deposit of levels 7, 6 and 5b, supporting the claim that the Early Middle Palaeolithic emerged in Europe about 300,000 years ago. 

## Materials and Methods

### U/Th dating

At Orgnac 3, two speleothems of well-crystallized calcite were collected from levels 7, 6 and 5b (Figure **4** and Figure **5**). The first speleothem, in levels 6 and 5b, is composed of two pieces of flowstones, each about 10 cm thick (Figure **5**). The samples PL1-1 and PL1-2, PL1-2a were taken from the upper part and PL1-3, PL1-4, PL1-4a and PL1-5 from the lower part of the upper piece. One sample PL1-6 was taken from the upper part of the lower piece (Figure **5**). Six more samples, PL2-1, PL2-2, PL2-3, PL2-4, PL2-4a and PL2-5 were taken from the top, the middle and the bottom, respectively, of a 46 cm-long stalagmite from levels 7 and 6 (Figure **4**).

The selected bulk subsamples were physically cleaned with ultrasonic methods [30]. U/Th chemistry was conducted in a class-10,000 metal-free clean room with class-100 benches at the High-precision Mass Spectrometry and Environment Change Laboratory (HISPEC), Department of Geosciences, National Taiwan University [30,31]. U-Th isotopic compositions and concentrations were determined on a sector-field inductively coupled plasma mass spectrometer (SF-ICP-MS), Thermo Fisher ELEMENT II [17] or a multi-collector ICP-MS (MC-ICPMS), Thermo Fisher NEPTUNE, with a dry introduction system, Cetac ARIDUS [16]. Uncertainties in all ICP-MS U/Th isotopic data were calculated at 2σ level and include corrections for procedure blanks, multiplier dark noise, abundance sensitivity, mass discrimination, and the occurrence of isotopes of interest in spike solution. Age was off-line calculated [17] with decay constants of 9.1705 × 10^-6^ yr ^-1^ for ^230^Th and 2.8221 × 10^-6^ yr ^-1^ for ^234^U [19], and 1.55125 × 10^-10^ yr ^-1^ for ^238^U [20].

### ^40^Ar/^39^Ar dating

Volcanic sediment samples were collected from level 2, about 1m below the top of the depositional sequence (Figure **4**). As sanidine is a proven chronometer [32], the largest possible and well-preserved sanidine grains (200-300 µm) were extracted using standard heavy liquid methods and then hand picked under a binocular microscope (Figure **7**). The obtained sanidine grains are angular and quite well preserved. Their chemical composition was estimated using scanning electron microscopy (Figure **7**) with Energy Dispersive X-ray Spectroscopy (EDS) in order to check the homogeneous presence of potassium (Ecole des Mines, Sophia Antipolis, Valbonne, France). The samples were irradiated for 40 minutes with Cd shielding in 5C position at McMaster University Reactor (Hamilton, Canada) and stored for one month at the Geoazur Laboratory (Nice, France). The sanidine grains were subsequently loaded onto a copper plate by sets of about 50-150 grains per hole for multigrain aliquot analyses (Figure **7**). Gas was extracted with an infrared continuous laser and purified in stainless and glass extraction line using two Al-Zr getters and a N_2_ cold trap. System blanks were run for every two or three analyzed samples. The mass spectrometer is a VG 3600 with a Daly detector. Mass discrimination was monitored by regularly analyzing one air pipette volume. The ultimate accuracy of the ^40^Ar/^39^Ar method depends on well-dated homogeneous standards [15,23]. J values were calculated using an age of 1.194 Ma [22] for ACS and the total decay constant of [33]. Recent revisions of decay and monitor constants suggest values about 0.6% ([15]; 1.201 Ma for ACS) and 1.0% ([34]; 1.206 Ma for ACS) older than those used here [35]. The implied difference in age is negligible for our samples (about 3 ka), therefore we use the conventional value of 1.194 Ma for ACS for all the ages. 
